# Extracellular Proteome Analysis and Flavor Formation During Soy Sauce Fermentation

**DOI:** 10.3389/fmicb.2018.01872

**Published:** 2018-08-15

**Authors:** Guozhong Zhao, Li-Li Ding, Yunping Yao, Yanping Cao, Zhi-Hui Pan, De-Hua Kong

**Affiliations:** ^1^State Key Laboratory of Food Nutrition and Safety, Key Laboratory of Food Nutrition and Safety, Ministry of Education of China, International Collaborative Research Center for Health Biotechnology, College of Food Engineering and Biotechnology, Tianjin University of Science & Technology, Tianjin, China; ^2^Beijing Laboratory for Food Quality and Safety, Beijing Technology and Business University, Beijing, China; ^3^Guangzhou Jammy Chai Sauce Workshop Co., Ltd., Guangzhou, China

**Keywords:** *Aspergillus oryzae*, soy sauce, proteome, protease, amylolytic enzymes, flavors

## Abstract

*Aspergillus oryzae* is an excellent strain for soy sauce fermentation because of its complicated enzyme system, especially protease. The aim of this study was to investigate the key enzymes and flavors during soy sauce fermentation, and a comparative assessment of extracellular enzymes during various fermentation stages at the proteomic level via iTRAQ analysis is presented. Many important enzymes related to the amino acid and glucose metabolisms participated in the material decomposition under high-salt stress. Dipeptidase, dipeptidyl aminopeptidase, leucine aminopeptidase, aspartic protease pep1, and extracellular metalloproteinase played positive roles during the early stage of soybean mash fermentation, whilst leucine aminopeptidase A and extracellular metalloproteinase NpI were the dominant proteolytic enzymes during the later period of fermentation. At the same time, β-glucosidase and β-xylanase exerted great effects upon glucose metabolism throughout the fermentation process. The results show that protease and amylolytic enzymes are complementary in the formation of flavors such as alcohols, acids, esters, aldehydes, furans, and pyrazines during soy sauce fermentation.

## Introduction

Soy sauce is the most popular of the traditional fermented soy foods such as miso, tempeh, natto, tofu, and soymilk products, in Asian cuisine worldwide. Soy sauce can enhance the secretion of gastric juice in humans and promote digestion (Kataoka, 2005). Soy sauce is made from a mixture of soybeans and wheat using an established two-step fermentation process, koji and soybean mash (brine) fermentation (Lioe et al., 2007). Soybeans and wheat at a ratio of about 1:1 are digested by *Aspergillus oryzae* to make koji, and about 18% sodium chloride is then added for fermentation for 6 months. Soy sauce is an indispensable condiment that is used for cooking in almost every household in China for its unique and abundant flavors. Soy sauce flavors depend on the raw materials and strains of microorganism used (Wanakhachornkrai and Lertsiri, 2003). The flavors come from the degradation of the available materials and the reaction of the degraded products during soy sauce fermentation, and nearly 300 compounds have been identified (Steinhaus and Schieberle, 2007).

*Aspergillus oryzae* is used for soy sauce fermentation and soybeans are the best nitrogen source for its growth. Various nitrogen sources greatly influence the extracellular production of protease (Hajji et al., 2008). *A. oryzae* can produce protease and amylolytic enzymes simultaneously and improve the flavor formation and nutritional quality of soybeans. The genome of *A. oryzae* contains 135 secreted protease genes, several amylolytic enzymes such as α-amylase and glucoamylase genes, which are differentially expressed during soy sauce fermentation (Machida et al., 2005). These proteases can be classified into alkaline, neutral, and acid proteases on the basis of the optimum pH. Proteases including proteinases and peptidases (endopeptidases, aminopeptidases, dipeptidases, and tripeptidases) play specific roles in the successive steps of protein degradation (Visser, 1993). Protease not only reduces the bitterness of soybean protein, but also determines the taste of the soy sauce because it cleaves proteins in soybeans to liberate amino acids or peptides and contributes correspondingly to an increase in the total nitrogen concentrations in the soluble fraction (Kitano et al., 2002; Sandhya et al., 2005). In addition, L-glutamine can be converted into L-glutamic acid, which enhances umami taste with glutaminase (Yuzuki et al., 2015).

*Aspergillus oryzae* secretes many hydrolases during koji fermentation of soy sauce. However, physical factors, such as the pH and the soybean mash concentration, decrease the hydrolase activity during soybean mash fermentation of soy sauce (Chutmanop et al., 2008). The protease is labile in brine solution, and the residual protease activity in an 18% NaCl solution is only about 3% (Su et al., 2005).

This study presents a comparative assessment of extracellular hydrolases during various stages of soy sauce fermentation at the proteomic level using the iTRAQ method. The flavors produced during soy sauce fermentation were also compared. The analysis revealed several hydrolases that play important roles in the degradation of materials and flavor formation.

## Materials and Methods

### Strain and Materials

*Aspergillus oryzae* 3.042 was isolated primarily from soil samples by the Shanghai Brewing Science Research Institute; it is widely used for soy sauce fermentation in China because it produces plenty of neutral and alkaline proteases and grows well in harsh environments. The soy beans and wheat bran in this study are organic, and come from Dongbei Province.

### Soy Sauce Fermentation

The koji medium was composed of 1 kg of the dregs of beans, steamed with 2.4 kg of hot water for 30 min and 1 kg of wheat bran and autoclaved at 120°C for 30 min. Spores of *A. oryzae* 3.042 were inoculated in the koji medium at 30°C for 24 to 30 h. At 12 and 18 h of cultivation, the medium was mixed again for aeration (Zhao et al., 2012), and 2- to 2.5-fold brine (18% NaCl) was then added for 6 months’ brine fermentation. The culture temperature was raised slowly from 16 to 30°C over 15 days (raise 1°C every day) during the early stage and maintained at 30°C for 6 months’ fermentation. Samples of soybean mash were collected during seven time periods: 0, 1, 2, 3, 4, 5, and 6 months. The samples were stored at -8°C immediately after collection.

### Extraction of Proteins for Proteomics Analysis

The samples were mixed with normal saline solution (1:2, m/m) at 40°C for 1 h in a water bath with stirring. The suspensions were filtered through a membrane filter with a 0.45-μm pore size. Ammonium sulfate powder was added, and the mixture was gently vortexed for 10 min. The concentration of ammonium sulfate was increased from 10 to 90%. After 24 h on ice, the mixture was centrifuged at 10,000*g* for 10 min at 4°C. The supernatant was removed, and the sediment was air-dried and suspended in 1 mL Tris–HCl solution (0.02 M, pH 7.5) (Jiang et al., 2004). The suspension was dialyzed against Tris–HCl solution (0.02 M, pH 7.5), and protease inhibitor phenylmethane sulfonyl fluoride (1 mM, 20 μg/mL) was added. The concentration of each protein sample was quantitated with the BCA Protein Assay Kit (Pierce) (Vanderschuren et al., 2014).

### TMT Labeling and LC-MS/MS Analysis

The peptides were desalted by Strata X C18 SPE column (Phenomenex) and vacuum-dried after the digestion of trypsin, then reconstituted in 0.5 M TEAB and processed according to the protocol of manufacturer for 6-plex TMT kit. One unit of 2,5-Dihydro-2,4,5-Trimethylthiazoline (TMT) reagent (label 100 μg of protein) was thawed and reconstituted in 24 μl acetonitrile (CAN).

HPLC Fractionation was carried out by the method of Wu et al. (2016). The samples were fractionated into fractions by high pH reverse-phase HPLC using Agilent 300 Extend C18 column (5 μm particles, 4.6 mm ID, 250 mm length). The peptides were dissolved in 0.1% of the formic acid (FA), directly loaded onto a reversed-phase pre-column (Acclaim PepMap 100, Thermo Scientific). Peptide separation was performed using a reversed-phase analytical column (Acclaim PepMap RSLC, Thermo Scientific). The gradient was comprised of an increase from 7 to 22% solvent (0.1% FA in 98% ACN) over 26 min, 22% to 35% in 8 min, and climbed to 80% in 3 min, then held at 80% for the last 3 min, all at a constant flow rate of 300 nl/min on an EASY-nLC 1000 UPLC system. The resulted peptides were analyzed by Q Exactive^TM^ plus hybrid quadrupole-Orbitrap mass spectrometer (Thermo Fisher Scientific).

The peptides were subjected to NSI source followed by tandem mass spectrometry (MS/MS) in Q Exactive^TM^ plus (Thermo) coupled online to the UPLC and selected for MS/MS using normalized collision energy (NCE) setting as 30; ion fragments were detected in the Orbitrap at a resolution of 17,500. A data-dependent procedure that alternated between one MS scan followed by 20 MS/MS scans was applied for the top 20 precursor ions above a threshold ion count of 10000 in the MS survey scan with 30.0 s dynamic exclusion. The electrospray voltage applied was 2.0 kV. Automatic gain control (AGC) was used to prevent overfilling of the orbitrap; 5E4 ions were accumulated for generation of MS/MS spectra. For MS scans, the m/z scan range was 350–1800. Fixed first mass was set as 100 m/z. Tandem mass spectra was searched against *Uniprot A. oryzae* and *Glycine max* database.

### Proximate Analysis

The contents of total nitrogen and reducing sugar in samples were determined according to the Kjeldahl method. Amino acid nitrogen and total acids were measured with the titration method (Jiang et al., 2007). The diluted samples (20 mL) were mixed with 60 mL H_2_O and titrated to pH 9.6 with 0.05 M NaOH before the addition of 37% formalin solution (10 mL). The consumed volume of NaOH was recorded to use in calculation of the total titratable acids of samples.

### Gas Chromatography-Mass Spectrometry Experiment

The soy sauce samples (5 mL) were extracted three times via head-space solid-phase micro-extraction (50/30 μm DVB/CAR-PDMS) at 45°C for 30 min in a water bath. The gas chromatograph mass spectrometer (Varian, Walnut Creek, CA, United States) was equipped with a VF-5-ms capillary column (30 m × 0.25 mm, 0.25 μm). The experimental procedure and mass spectra comparison of gas chromatography-mass spectrometry was explored previously (Zhao et al., 2015). 2-octanol was used as the internal standardization in this study.

## Results and Discussion

### Changes in Soy Proteins During Soy Sauce Fermentation by Proteomics Analysis

**FIGURE 1:**
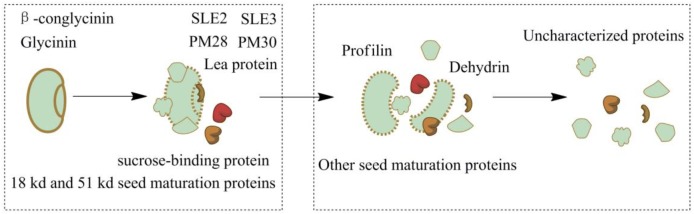
Variations of soy proteins during soy sauce koji fermentation and soybean mash fermentation.

Soybeans, one of the main ingredients in soy sauce, contain 36 to 56% protein. Glycinin and β-conglycinin accounted for 65 to 80% of the total soy proteins (Singh et al., 2014). Many soy proteins are broken down during the first stage (soy sauce koji fermentation), such as some uncharacterized proteins, glycinin, glycinin G2 and the 18 kDa seed maturation protein (**Supplementary Tables S1, S2**). The relative amounts of other proteins were increased at the same time because of the continuous hydrolysis. Protein SLE3, protein SLE2, 18 kDa seed maturation protein, 51 kDa seed maturation protein, seed maturation protein PM28, seed maturation protein PM30, sucrose-binding protein and lea protein were difficult to destroy under submerged conditions, but they were decomposed much easier via solid-state koji fermentation. Profilin, dehydrin, and other seed maturation proteins gradually disappeared during the 180 days of soybean mash fermentation. Some uncharacterized proteins remained untill the fermentation end-point because of their complex and stable protein structures (**Figure 1**). These changes in the soy proteins should be attributed to proteolysis of various kinds of protease. The results show that *A. oryzae* had the best hydrolysis effect during soy sauce fermentation.

### Hydrolase Identification, Quantification, and Analysis

To identify the fermentation mechanism of soy sauce, we elucidated the quantitative changes in the proteome of extracellular hydrolases during koji fermentation and during the early, medium and late stages of soybean mash fermentation. Dozens of up-regulated and down-regulated hydrolases were revealed by processing the data. We found that these hydrolases were mostly proteases and amylolytic enzymes (**Table 1**).

**Table 1:** Extracellular hydrolases of *A. oryzae* quantified with greater than 1.5-fold changes.

Gene ID	Protein description	Koji		Soybean mash	
		4S/1S	1L/3L	3L/6L
Ao3042_08984	Dipeptidase	1.33	1.69	/
Ao3042_04957	Dipeptidyl aminopeptidase	1.68	3.66	/
Ao3042_09175	Puromycin-sensitive aminopeptidase	1.73	/	/
Ao3042_02016	Xaa-pro aminopeptidase	1.88	/	/
Ao3042_06191	Neutral protease 2	2.57	/	/
Ao3042_08351	Neutral protease 2	4.27	0.20	5.09
Ao3042_10620	Leucine aminopeptidase 2	1.60	5.41	/
Ao3042_09696	Tripeptidyl-peptidase sed2	0.36	/	/
Ao3042_02733	Alkaline protease 1	/	0.05	/
Ao3042_09529	Extracellular metalloproteinase	/	16.35	0.85
Ao3042_09542	Leucine aminopeptidase A	/	2.15	0.59
Ao3042_04093	Aspartic protease pep1	/	1.56	/
Ao3042_10648	Glucoamylase	1.33	/	/
Ao3042_01015	β-glucosidase	1.42	3.62	/
Ao3042_02876	β-glucosidase-related glycosidase	2.02	/	/
Ao3042_04282	Glucan endo-1,3-β-glucosidase eglC	1.73	/	/
Ao3042_10248	β-xylanase	2.46	26.63	/
Ao3042_06121	β-xylanase	2.01	2.33	/
Ao3042_06920	β-xylanase	2.57	/	/
Ao3042_09482	Hydrolytic enzyme of the α/β hydrolase fold protein	1.85	/	/
Ao3042_06922	α-L-arabinofuranosidase axhA	0.38	/	/
Ao3042_02530	Putative unsaturated glucuronyl hydrolase	0.42	/	/
Ao3042_04009	Endo-chitosanase	0.21	/	/

*4S/1S, samples cultured in solid-state culture for koji fermentation about 24 h (4S)/18 h (1S); 1L/3L, samples cultured in submerged culture for soybean mash fermentation about 1 month (1L)/3 month (3L); 3L/6L, samples cultured in submerged culture for soybean mash fermentation about 3 month (3L)/6 month (6L)*.

Most hydrolases were identified in soy sauce koji (**Supplementary Tables S3, S4**). A broad range of proteinases, such as peptide hydrolase, dipeptidase, aminopeptidase, and neutral protease, were compared and were found to be up-regulated more in 4S than 1S, but tripeptidyl peptidase sed 2 was down-regulated in 4S more than two times. Tripeptidyl peptidase was secreted in the early stage during koji fermentation. Endopeptidases (asparaginyl endopeptidase) and exopeptidases (aminopeptidase, carboxypeptidase, dipeptidase, dipeptidyl peptidase) are involved in the metabolism of soybean proteins. Endopeptidases specifically cleave peptide bonds C-terminal to amino acids (Saska et al., 2007). Aminopeptidases specifically cleave the amino terminal residue from polypeptide chains; for example, the leucine amino peptidase can remove N-terminal leucine from peptide chain (Nampoothiri et al., 2005). The expression level of neutral protease in 4S was increased by more than twice in 1S. Neutral protease cleaves soy protein into peptides. Dipeptidase, peptide hydrolase, and aminopeptidase are then involved in the further degradation of small peptides. Dipeptidase, dipeptidyl aminopeptidase, leucine aminopeptidase, aspartic protease pep1, and extracellular metalloproteinase play positive roles during the early stage of soybean mash fermentation, whilst leucine aminopeptidase A and extracellular metalloproteinase NpI are the dominant proteolytic enzymes during the later period of fermentation. β-glucosidase and β-xylanase, which are essential for the complete hydrolysis of cellulose to glucose, decreased significantly during soybean mash fermentation (Bai et al., 2013). **Table 1** shows that some hydrolases were autolysed during soybean mash fermentation. A few type of hydrolase still existed during the late stage of soybean mash fermentation.

### Proximate Analysis During Soybean Mash Fermentation

The content of total nitrogen was increased from 0.87 ± 0.04 g/100 g to 1.59 ± 0.06 g/100 g after 60 days of soybean mash fermentation, depending on the proteases of *A. oryzae*. Peptides and free amino acids were generated from soy proteins via digestion with the proteases that could catalyze the hydrolysis of peptide bonds. The concentration of amino acid nitrogen was increased from 0.44 ± 0.02 g/100 mL to 0.93 ± 0.03 g/100 mL after 60 days of fermentation; it decreased thereafter for the remaining 120 days of fermentation. The value of the amino acid nitrogen represents the total free amino acids.

**FIGURE 2:**
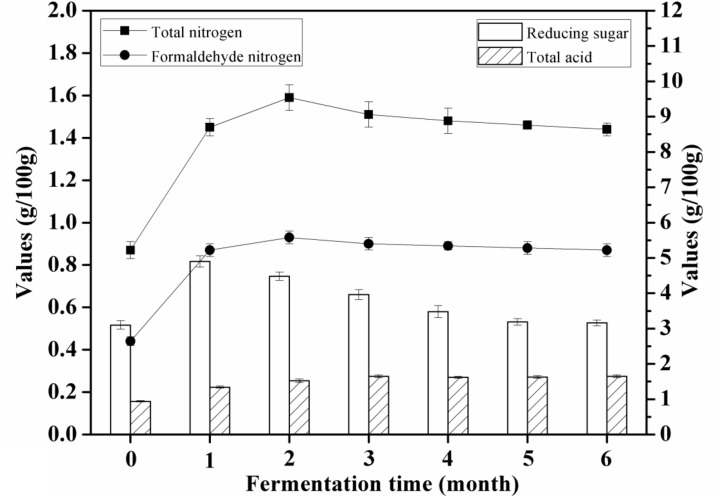
Changes in proximate indices (total nitrogen, amino acid nitrogen, reducing sugar, and total acid) of soybean mash samples during brine fermentation.

Reducing sugar increased to 4.94 ± 0.16 g/100 g after 30 days of fermentation and then decreased to 3.19 ± 0.09 g/100 g after the remaining 150 days. Glycoside hydrolases play a significant role in producing the reducing sugar during the initial brine fermentation. The content of total acids increased significantly throughout the fermentation period (**Figure 2**). The increase in total acids content of the soy sauce may be as a result of the tricarboxylic acid (TCA) cycle during soy sauce fermentation.

### Flavor Formation During Soybean Mash Fermentation

The flavor produced during fermentation is a very important factor in soy sauce, mainly due to the hydrolysis of the protein, starch and other macromolecules to a variety of secondary products and small molecular final products with the action of enzymes secreted by *A. oryzae*. These secondary products react with a series of biochemical reactions, including protein hydrolysis, alcohol fermentation, organic acid fermentation, and lipid formation to form the nutrients and flavor substances. Moreover, the abundance of alcohols, organic acids, and esters, especially ethanol, acetic acid, lactic acid, and glycerol, increase the flavor of traditional fermented soy sauce.

**FIGURE 3:**
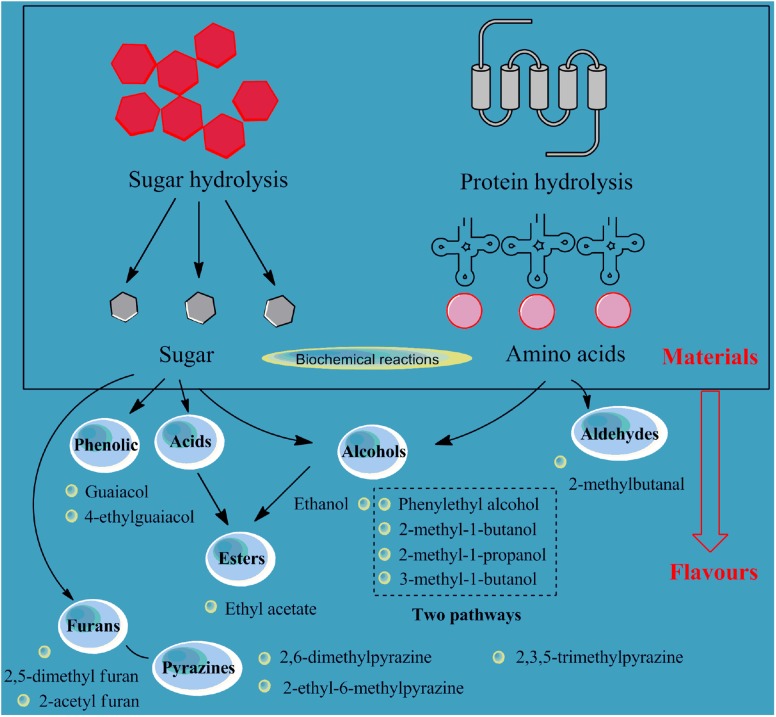
Relationship of the flavors (alcohols, acids, aldehydes, esters, phenolic, furans, and pyrazines) and the hydrolysis of protein and starch materials for soy sauce fermentation.

The flavors of soy sauce samples at the end of the fermentation process were determined in this study with head-space solid-phase micro-extraction combined with gas chromatography-mass spectrometry. As shown in **Supplementary Table S5**, 85 volatiles were detected, including 18 alcohols and 74.86% of the total content (13 esters, 5.37%; 5 acids, 1.65%; 14 ketones, 4.32%; 15 pyrazines, 7.46%; 2 phenols, 1.52%; 6 aldehydes, 2.47%; 2 furans, 0.33%; and 10 heterocycles, 2.02%). In summary, alcohols, ketones, esters, and pyrazines were the main components of the flavors of the soy sauce samples, followed by aldehydes, acids, phenols, and furans (**Figure 3**).

In detail, alcohol was the largest group of flavors. Some kinds of alcohols in soy sauce samples are formed by the conversion of aldehydes and some by the reaction of amino acids and sugars under aerobic conditions (Shu et al., 2012). The soy sauce samples’ contents of phenylethyl alcohol, ethanol, 3-methyl-1-butanol, 2-methyl-1-butanol, and 2-methyl-1-propanol are higher than those of other alcohols. Two pathways are used to produce polyols such as phenylethyl alcohol, 3-methyl butanol, 2-methyl butanol, and 2-methyl propanol. One pathway involves the degradation of amino acids such as phenylalanine, leucine and isoleucine, and the other involves the processes of carbohydrate metabolism and amino acid biosynthesis (Feng et al., 2013).

Ester is another important aroma compound in soy sauce due to the high volatility and sensitivity to human olfactory receptors. Most esters are derived from esterification and dehydration of alcohols and acids under the action of esterification enzymes during the fermentation process and are influenced by the contents of precursor acids and alcohols in the matrix. Most ethyl esters that may be related to the high concentration of ethanol produced during fermentation can impart a fruity, sweet or floral odor to the soy sauce. Ethyl acetate is the most common ester in soy sauce, and the content measured in this study was about 62.6 μg/L.

The aldehydes and ketones in soy sauce accelerated the appearance of pleasing aromas, such as sweet, grassy, fruity, and charred. The content of 2-methylbutanal, a kind of carbonyl compound, was 30.4 μg/L, and it can be degraded by amino acids. Therefore, the production of this substance may be related to the metabolism of soy proteins in the fermentation process.

Phenolic, pyrazine, and furan compounds are very important compounds in fermented soy products. Pyrazines are come from the Strecker degradation of α-amino acids and reductones (Xiao et al., 2015). Furans are probably come from the degradation of glucose, a thermal degradation product of cellulose (Toldrá and Flores, 2006). The two phenolic substances detected in this study were guaiacol and 4-ethylguaiacol, which are the two most common flavor compounds in soy sauce and could contribute to the smoky aroma. These phenolic substances are often considered to be related to the metabolism of lignin. Studies have shown that increasing the proportion of wheat flour in the raw material can increase the content of such substances (Nunomura and Sasaki, 1992).

For pyrazines, 2,6-dimethylpyrazine, 2-ethyl-6-methylpyrazine, and 2,3,5-trimethylpyrazine (68.6, 31.9, and 26.5 ug/L, respectively) were detected in the samples. These substances were also detected in cooked soybeans and other fermented soy products (such as natto and soybean paste) (Lee and Ahn, 2009). Three furan compounds were detected in the samples: furfuryl alcohol, 2,5-dimethyl furan, and 2-acetyl furan. The content of furfuryl alcohol was 98.5 μg/L. 2-acetyl furan is believed to have a caramel-like aroma and is generally produced during the sugar degradation process in the Maillard reaction (Mottram, 2007).

## Conclusion

*Aspergillus oryzae* plays an important role during fermentation. The hydrolases, especially the proteases and the glycoside hydrolases, were secreted during koji fermentation. The kinds of hydrolases created during soy sauce fermentation were identified in this study. Soy protein and wheat powder were decomposed by the actions of these hydrolases, but some uncharacterized proteins remained until the fermentation end-point for their complex and stable structures. The expression levels of hydrolases such as dipeptidase, neutral protease 2, leucine aminopeptidase, and β-xylanase were not constant and decreased gradually throughout soy sauce fermentation. We also found that some flavors were connected with the hydrolytic amino acids and reducing sugar. These findings have important implications for soy sauce fermentation.

## Author Contributions

GZ and YY conceived the initial project idea. L-LD and Z-HP designed the approach to be used. L-LD and Z-HP generated the laboratory data. D-HK, YY, and YC collated, analyzed, and interpreted the data. GZ and L-LD wrote the paper. All authors provided comment on the manuscript prior to submission.

## Conflict of Interest Statement

The authors Z-HP and D-HK were employed by company Guangzhou Jammy Chai Sauce Workshop Co., Ltd. The remaining authors declare that the research was conducted in the absence of any commercial or financial relationships that could be construed as a potential conflict of interest.
